# Editorial: The spectrum of eating behaviours in athletes: health and performance consequences

**DOI:** 10.3389/fnut.2026.2021497

**Published:** 2026-10-20

**Authors:** Heather Hower, Suzanne Dooley-Hash, Morteza Homayounnia Firouzjah

**Affiliations:** 1 Department of Psychiatry and Human Behavior, Warren Alpert Medical School, Brown University, Providence, RI, United States; 2 Department of Health Services, School of Public Health, Policy, and Practice, Brown University, Providence, RI, United States; 3 Department of Psychiatry, Medical School, University of California, San Diego, San Diego, CA, United States; 4 Department of Emergency Medicine, Medical School, University of Michigan, Ann Arbor, MI, United States; 5 Department of Physical Education, Farhangian University, Tehran, Iran

**Keywords:** anorexia, athlete, body image, bulimia, eating disorders, exercise, nutrition, Relative Energy Deficiency in Sport (RED-S)

Eating disorders (ED) such as anorexia nervosa (AN), bulimia nervosa (BN), and binge eating disorder (BED), as defined in the “*Diagnostic and Statistical Manual of Mental Disorders-5* (DSM-5) (1), are increasingly observed among athletes, affecting their health, wellbeing, and career longevity (2). Significantly more prevalent in the athletic community than in the general population (3), ED are linked to the higher physical demands and aesthetic pressures prevalent in certain sports. Particularly in sports where weight control is crucial for performance or appearance—such as gymnastics, ballet, wresting, and long-distance running—athletes face heightened risks (4). These conditions not only endanger athletes' health, but also potentially truncate their professional careers, emphasizing the importance of effective preventative and intervention strategies (5).

This Research Topic aimed to explore how competitive sports contribute to ED behaviors and nutritional issues among athletes, delve into the root causes of ED in these environments, and propose actionable, evidence-based interventions. The goal was an analysis covering a wide spectrum of sports and demographics, with clinical implications for reducing both the prevalence and the impact of ED. To foster a more comprehensive understanding, this Research Topic embraced contributions that addressed several critical aspects, including: studies examining the prevalence of ED among diverse sports disciplines and athlete populations; comparative research detailing differences in nutritional health between elite athletes and their non-elite counterparts; investigations into the direct effects of specific ED on physical performance and recovery; analysis of both risk and protective factors in athlete populations, with a focus on creating resilience strategies; research that offers new perspectives or solutions for managing ED in athletes; and guidelines for athletic and health professionals to foster healthier environments.

Within this Research Topic, Woronyczová et al. underscored the severity of Relative Energy Deficiency in Sport (RED-S) related to long-term low-calorie intake in athletes; low energy availability (LEA). Compared to healthy control participants, Czech female (ages 15–30) endurance athletes demonstrated significant deficits in energy availability, lower serum concentrations of essential hormones, and greater menstrual irregularity (e.g., amenorrhea).

In examining a different population of Chinese male adolescent combat sport athletes, Meng et al. found that the majority of participants engaged in intentional weight loss practices (e.g., increased exercise, wearing plastic suits to sweat in) for over 2 weeks before their weigh-in for the athletic event. Most participants reported that they believed that their lighter weight would be beneficial for their athletic performance, and would have no impact on health.

In a further exploration of adolescents' experiences with navigating the intersection of their gender, sport, and dietary identities, Deslippe et al. interviewed a diverse cohort of high school athletes and non-athlete controls, utilizing an interpretive phenomenological approach. A prominent theme that emerged centered around eating behaviors as a demonstration of identities (e.g., boys eating protein to develop muscle, girls taking smaller bites to remain thin). However, the adolescents also held conflicting views about masculinity (toxic but also useful in competition).

These studies highlight the current challenges and intervention considerations in working with athletes with ED. Vasiliu contributed a synthesis of available data regarding a specific pathology reported in elite athletes (anorexia athletica) based on a narrative reviewing methodology. While tentative diagnostic criteria and a self-administered questionnaire exist, further research on its pathophysiology is needed, particularly in subpopulations of elite athletes at high risk (e.g., in aesthetic, individual sports, with a prior history of an ED).

A promising clinical intervention for the above-noted concerns was studied in an athlete-focused ED program in higher levels of care (partial hospitalization and intensive outpatient) by Dunbar et al. Participants included 182 adolescent and adult athlete patients who were assessed for dysfunctional exercise symptoms, ED symptoms, related impairment, depression, and anxiety at admission, mid-treatment, discharge, and 6- and 12-month follow-ups. Significant decreases were observed across all assessment measures, with outcomes showing stabilization through the 12-month follow-up. The findings provide preliminary empirical support for the feasibility and clinical utility of programming that addresses the challenges and needs of athletes.

In summary, the results of the above mentioned studies and reviews represent a range of new perspectives on the spectrum of ED in athletes, and implications for health and performance. Indeed, the Research Topic covers diverse sports disciplines (e.g., aesthetic and non-aesthetic, elite and non-elite) and athlete populations (e.g., adolescents-adults, all sexes/genders, residing in the community-higher levels of care, from five different countries). Each contribution addressed the prevalence, risk factors, impact of ED in populations of athletes, and implications for further research and clinical intervention areas. Studies which provide potential new solutions for this unique population offer hope for prevention, early intervention, and more tailored treatments.
